# Quantitative phenotypic and pathway profiling guides rational drug combination strategies

**DOI:** 10.3389/fphar.2014.00118

**Published:** 2014-05-28

**Authors:** John C. Dawson, Neil O. Carragher

**Affiliations:** Edinburgh Cancer Discovery Unit, Edinburgh Cancer Research UK Centre, MRC Institute of Genetics and Molecular Medicine, University of EdinburghEdinburgh, UK

**Keywords:** phenotypic screening, drug combinations, high-content, proteomics, network pharmacology

## Abstract

Advances in target-based drug discovery strategies have enabled drug discovery groups in academia and industry to become very effective at generating molecules that are potent and selective against single targets. However, it has become apparent from disappointing results in recent clinical trials that a major challenge to the development of successful targeted therapies for treating complex multifactorial diseases is overcoming heterogeneity in target mechanism among patients and inherent or acquired drug resistance. Consequently, reductionist target directed drug-discovery approaches are not appropriately tailored toward identifying and optimizing multi-targeted therapeutics or rational drug combinations for complex disease. In this article, we describe the application of emerging high-content phenotypic profiling and analysis tools to support robust evaluation of drug combination performance following dose-ratio matrix screening. We further describe how the incorporation of high-throughput reverse phase protein microarrays with phenotypic screening can provide rational drug combination hypotheses but also confirm the mechanism-of-action of novel drug combinations, to facilitate future preclinical and clinical development strategies.

## Introduction

The evolution of many complex human diseases has generated multiple biological redundancies in the genetics, pathway signaling networks and pathophysiology of disease thus counteracting the efficacy of new therapeutics. In such complex diseases exemplified by cancer, neurodegeneration, cardiovascular, bacterial, and viral infections, combination therapies represent the standard of care (Zimmermann et al., 2007; Al-Lazikani et al., 2012; Yap et al., 2013). Examples of fixed dose and co-administration drug combination therapies approved across multiple disease indications are described in Table 1.

**Table 1:** **Examples of drug combinations approved for clinical use across disease indications**.

**Indication**	**Approved drug combinations**
Melanoma	Trametinib (a MEK inhibitor) + Dabrafenib (a BRAF inhibitor) approved through the FDA accelerated approval program (Flaherty et al., 2012)
Pancreatic cancer	Gemcitabine + nanoparticle albumin-bound nab-paclitaxel (Abraxane) (Sahoo and Kumar, 2014; Saltz and Bach, 2014; Von Hoff et al., 2014); FOLFIRINOX (5-Fluorauracil + leucovorin [Wellcovorin] + irinotecan [Camptosar] + oxaliplatin) (Oikonomopoulos et al., 2014)
Ovarian cancer	Taxane/platinum combination therapy (carboplatinum/paclitaxel) represents standard of care
Breast cancer	Multiple chemotherapeutic regimes include; Adriamycin + Cyclophosphamide; Adriamycin + Cyclophosphamide + Paclitaxel; Cyclophosphamide + Adriamycin + Fluorouracil; Cyclophosphamide + Methotrexate + Fluorouracil; Fluorouracil + Epirubicin Hydrochloride + cyclophosphamide; Taxotere + Adriamycin + cyclophosphamide
Diabetes	Xigduo (sodium-glucose linked transporter 2 [SGLT2] + metformin) (Jabbour et al., 2014)
Cardiovascular	Vytorin (Eztimibe + simvastatin), Caduet (Amlodipine + Atorvistatin), and Lotrel (Amlodipine + Benezapril) (Dabhadkar and Bellam, 2013)
Antiviral (HIV)	Multi-component anti-retroviral therapies include, Atripla (efavirenz + tenofovir/emtricitabine); Complera (rilpivirine + tenofovir/emtricitabine); and Stribild (elvitegravir + cobicistat + tenofovir/emtricitabine)
Antibacterial	Combinations targeted against antibacterial resistance include, Bactrim (Trimethprim + Sulfa mexathazole); B-Lactamase inhibitor *or* carbapenem + aminoglycoside (for gram negative infection); B-Lactam + streptogramin or teicoplanin + aminoglycoside (for vancomycin resistant gram negative infection) (Sacks and Behrman, 2009)

With regards to cancer it is apparent that further exploitation of new targeted therapies including novel combinations of unapproved agents and targeted therapy combined with established chemo- or radio-therapy remain to be fully explored in preclinical and clinical settings. Numerous clinical trials are progressing that promise to maximize the value of targeted drugs as combination therapies in cancer (Yap et al., 2013). The challenge is to select which of the many drug combination possibilities and administration schedules are most suitable for clinical development across distinct cancer patient populations.

Given the widespread use and strong track record of clinical success of combination therapy across complex diseases, it is surprising that most typical drug discovery strategies only consider drug combinations during late-stage development or as a risk mitigation strategy. The re-emergence of phenotypic drug discovery (PDD) strategies provides a new opportunity to discover and prioritize drug combination and polypharmacology strategies objectively while appropriately tailoring their use to complex disease during early stage drug discovery (Lee and Berg, 2013). In this review article we highlight how new advances in high-content screening and high throughput pathway profiling capabilities advance phenotypic screening approaches toward a more systematic discovery of the next generation of multi-targeted drugs and drug combination therapies.

## Phenotypic screening of drug combinations

Recent advances in automated image-based high-content microscopy provide a new opportunity to perform hypothesis-free phenotypic screening of complex compound libraries and drug combination sets in more sophisticated biological assays (Bickle, 2010). To support the discovery of novel combination therapies, experimental assay systems that maintain the integrity of biological signaling networks in their most physiologically relevant states are most desirable. In their simplest form, such phenotypic assays may represent single cell-based assays, however, more elaborate co-culture or 3-dimensional (3D) organotypic models can be employed to explore multi-targeted intervention of paracrine or juxtacrine signaling between distinct cell populations (Harma et al., 2010).

A wealth of preclinical drug combination data using cell based and *in vivo* models has been published that serves as the basis for clinical proof-of-concept studies and patent claims of novel drug combination strategies. A limitation of much of the patented and published drug combination studies to date is that they are often performed or presented as isolated studies focusing on a specific combination. As such, these combinations are not placed into context of broader combination options or benchmarked against standard-of-care therapies. The reductionist approaches to the study of specific combination therapies limits the ability of drug development and clinical research groups to objectively prioritize and iterate the most effective drug combination strategies to move forward into late stage preclinical or clinical development. Another major limitation of many preclinical drug combination studies is the physiological relevance of the findings. For example, the identification of synergistic activity at doses that are not achievable *in vivo* or at time points incompatible with the *in vivo* pharmacokinetic properties of the individual components of each combination are highly unlikely to succeed. Thus, advances in high-throughput phenotypic screens including, increased throughput, kinetic profiling drug response in live-cell systems, analysis of multiple phenotypic endpoints across potentially more relevant 3D and co-culture models facilitate a more comprehensive and transparent approach to both hypothesis-driven and hypothesis-free exploration of drug combinations.

The application of phenotypic drug combination screening is exemplified by dose-ratio matrix testing multiple pairwise combinations across cell based assays enabling analysis of synergy, additive, and antagonistic effects across diverse chemical libraries, annotated compound libraries and approved drug sets (Zimmermann et al., 2007). Recent examples of dedicated drug combination screening campaigns using a variety of phenotypic assays and distinct endpoints have been published (Axelrod et al., 2013; Cubitt et al., 2013; Du et al., 2013; Held et al., 2013; Schmidt et al., 2013; Li et al., 2014). Such phenotypic screens have identified novel synergistic combinations such as: Lapatanib (EGFR and Her2 inhibitor) combined with the multi-targeted inhibitor Ro31-8220 (Axelrod et al., 2013); Lapatanib combined with MK2206 (Akt inhibitor) (Held et al., 2013) and Rapamycin (mTOR) combined with Sunitinib (multi-targeted kinase inhibitor) (Li et al., 2014). For pragmatic reasons such recent examples of dedicated combination screening has been mostly limited to small focussed compound libraries and 2-dimensional (2D) cell based assays.

A limitation of screening large compound libraries in complex cell based assays compared with more traditional biochemical drug screening is throughput and cost. Both throughput and cost are particularly limiting when considering the evaluation of multiple drug combinations across a factorial dose-ratio matrix where the number of individual combination dose ratios increases quadratically with the number of agents under study. For practical reasons, medium to high-throughput phenotypic screening across cancer cell lines have traditionally employed simple single endpoint analysis of tumor cell viability or cell proliferation in 2D mono-culture (Barretina et al., 2012). While such assays can provide valuable insights into phenotypic and drug combination response across annotated cell line panels their reliance on gross cell viability and proliferation endpoints tend to favor the phenotypic discovery of cytotoxic agents. Furthermore, integration of basic cell viability endpoints with gene expression profiling provide a useful source of biomarkers that predict sensitivity to cell-cycle arrest but poorly inform on optimal combination strategies or markers for other important cancer phenotypes such as apoptosis and invasion. Recent advances in fully automated brightfield and fluorescent microscopic acquisition platforms and associated image analysis algorithms have facilitated the integration of quantitative microscopic imaging of multiple endpoints upon both fixed and live-cells assays (Perlman et al., 2004; Yarrow et al., 2004; Tanaka et al., 2005; Caie et al., 2010). Screening beyond simplistic 2D monoculture assays is a necessary aim to target more relevant pathophysiological mechanisms and discover novel synergistic drug combination activity. Drug combination screening in complex 3D and co-culture assay formats is most desirable, however, throughput is limiting when using standard cell culture assay methods especially for hypothesis-free screening. The integration of high-content microscopy platforms with sophisticated laboratory automation and optimized data-handling pipelines provide increased throughput and overcome many of the bottlenecks associated with screening large compound libraries across complex assay formats (Bickle, 2008; Alcock et al., 2010). The application of validated mathematical approaches, based upon the median-effect principal and combination index theorem (Chou and Talalay, 1981, 1984) provides robust evaluation of additive, synergy, and antagonism of drug combination effects. Further optimization of software tools specifically designed for the analysis and visualization of large drug combination screening data sets are based upon methods such as Lowe additivity (Zimmermann et al., 2007) and are exemplified by the *combination* high throughput screening (cHTS) platform (Zalicus®) and Compound Synergy Extension (Genedata Screener®). Such tools enable rapid drug combination screening across phenotypic assays at scale to enable a more transparent review of drug combination data placed into context of multiple drug combination sets to aid benchmarking and prioritization. Incorporation of genomically annotated patient derived cell panels into high capacity drug combination screening activity further supports pharmacogenomics and personalized healthcare approaches to drug combination strategies. High-content single cell analysis of specific phenotypic events over both dose and time enable a significantly more robust evaluation of the quality of drug combination data. Such considerations support the interpretation of whether synergistic and additive drug combination data occur at physiologically relevant doses.

A significant challenge to translating effective drug combinations identified from *in vitro* screens to *in vivo* models and the clinical setting is balancing the distinct pharmacokinetic properties of each individual component of a combination to ensure drug uptake and retention times within the target tissue *in vivo* replicates optimal synergistic dose ratios identified from *in vitro* phenotypic assays. Phenotypic screening of drug combinations across live cell kinetic assays provide further information on the optimal duration of time each component of a drug combination needs to be present together to provide synergistic activity. Kinetic phenotypic analysis is enhanced by image-based reporters of functional endpoints that are compatible with live cell assays as exemplified by the cell-permeable caspase 3 biosensor probe NucView™ which provides a real time readout of caspase activation and induction of apoptosis (Smith et al., 2012). By correlating the kinetics of apoptosis induction to drug combination treatment observed *in vitro* with the *in vivo* pharmacokinetic properties of the drugs, optimal *in vivo* scheduling of drug combinations can be predicted. Kinetic profiling of phenotypic response following drug combination treatment also allows selection of the most appropriate time-points to perform analysis of synergy and antagonistic activity. Building drug combination properties into single molecules such as a multi-targeted small molecule, bi-specific antibody or formulated fixed-dose combination product (polypill) circumvents many of the challenges associated with co-dosing distinct components *in vivo*. Thus, application of more systematic approaches to evaluate the kinetics and sensitivity of drug combinations across broad dose ranges and multiple phenotypic parameters promise to enhance the quality and robustness of preclinical drug combination data and support more informed prioritization of the most appropriate combination strategies to move forward into *in vivo* and clinical settings.

Despite recent advances in automated high-content microscopy, integrating such systematic approaches to drug combination screening in the more advanced preclinical models is always going to be limited by throughput. This is particularly the case when additional considerations such as sequencing of drug combination treatments and greater than pairwise drug combination cocktails are under consideration. Application of computational biology and unbiased artificial intelligence approaches to predicting and/or guiding drug combination selection may overcome the bottlenecks associated with empirical testing of every possible drug combination dose-ratio in both hypothesis-free and hypothesis-driven screening of combinations in complex *in vitro* or *in vivo* models (Lehar et al., 2009; Azmi et al., 2010). The application of genetic algorithms from the field of computational artificial intelligence has been used to guide the selection of drug combinations for empirical testing (Zinner et al., 2009). Zinner et al. employed a genetic algorithm approach to identify novel multi-drug cocktails effective upon cancer cell line proliferation. Using a “fitness function” parameter, defined by pharmacological performance in a cell proliferation assay, the most effective combinations from first generation testing of a small combination set was used to guide algorithmic selection of subsequent generations of combinations representing a sample of a larger compound library. Iterative rounds of testing and selection of the “fittest” combinations provide a rational sampling approach of broad areas of drug combination space. The multi-drug cocktail, Feretinied, suberoylanide hydroxyamic acid, and bortezomib was determined to be the fittest in the A549 non-small cell lung carcinoma (NSCLC) proliferation assay and enhanced efficacy and synergy was subsequently validated in other NSCLC cell lines (Zinner et al., 2009). The complexity of drug combinations would not be limiting if using a genetic algorithm approach, which iteratively samples a small proportion of the best drug combinations extracted from large compound libraries. This approach enables intuitive exploration of new chemical entities, multi-drug cocktails, and alternate drug combination sequencing strategies. The application of multiparametric high content assays that inform on both the efficacy and toxic liability of preclinical drug combinations may assist in defining a multiparametric fitness function that enables a genetic algorithm to direct a guided search of drug combination space toward efficacy and away from toxicity.

## Functional proteomics

A major challenge to the successful clinical application of targeted therapies is the existence of complex intrinsic and adaptive resistance mechanisms that have evolved to maintain the selective advantage of disease systems. While many of the underlying causes of disease occur at the genetic level, drug response and resistance are often governed by epigenetic and post-translational mechanisms. Recent studies indicate that disease pathogenesis, particularly for cancer is associated with the co-activation of multiple signaling pathways (Stommel et al., 2007; Duncan et al., 2012; Lee et al., 2012). Furthermore, targeted therapies specific for these signaling pathways reprogram signaling networks thus providing for multiple compensatory and redundancy mechanism (Duncan et al., 2012; Lee et al., 2012). Therefore, combinations of drugs will be most effective in treating such adaptive systems if we can elucidate the networks and pathway switching mechanisms that permit diseased cells to subvert single therapeutic agents. Intracellular and paracrine signaling events are highly dynamic and drugs influence the temporal dynamics of signaling networks highlighting the importance of studying signaling events temporally following drug exposure to provide rational for simultaneous or sequential drug combination strategies (Lee et al., 2012). Recent advances in the generation and interpretation of proteomic data complements genomic analysis by providing additional information on pathway activation states providing new insight into complex biochemical pathways driving disease mechanisms and controlling therapeutic response (Kolch and Pitt, 2010). New advances in high-throughput functional proteomics combined with phenotypic screening in more relevant and informative biological models may provide the necessary rationale for selecting drug combinations and for pairing biomarkers to inform on drug combination mechanism-of-action studies and patient stratification strategies for combination therapy.

### Reverse phase protein arrays: high-throughput chemical proteomics

**Figure 1:**
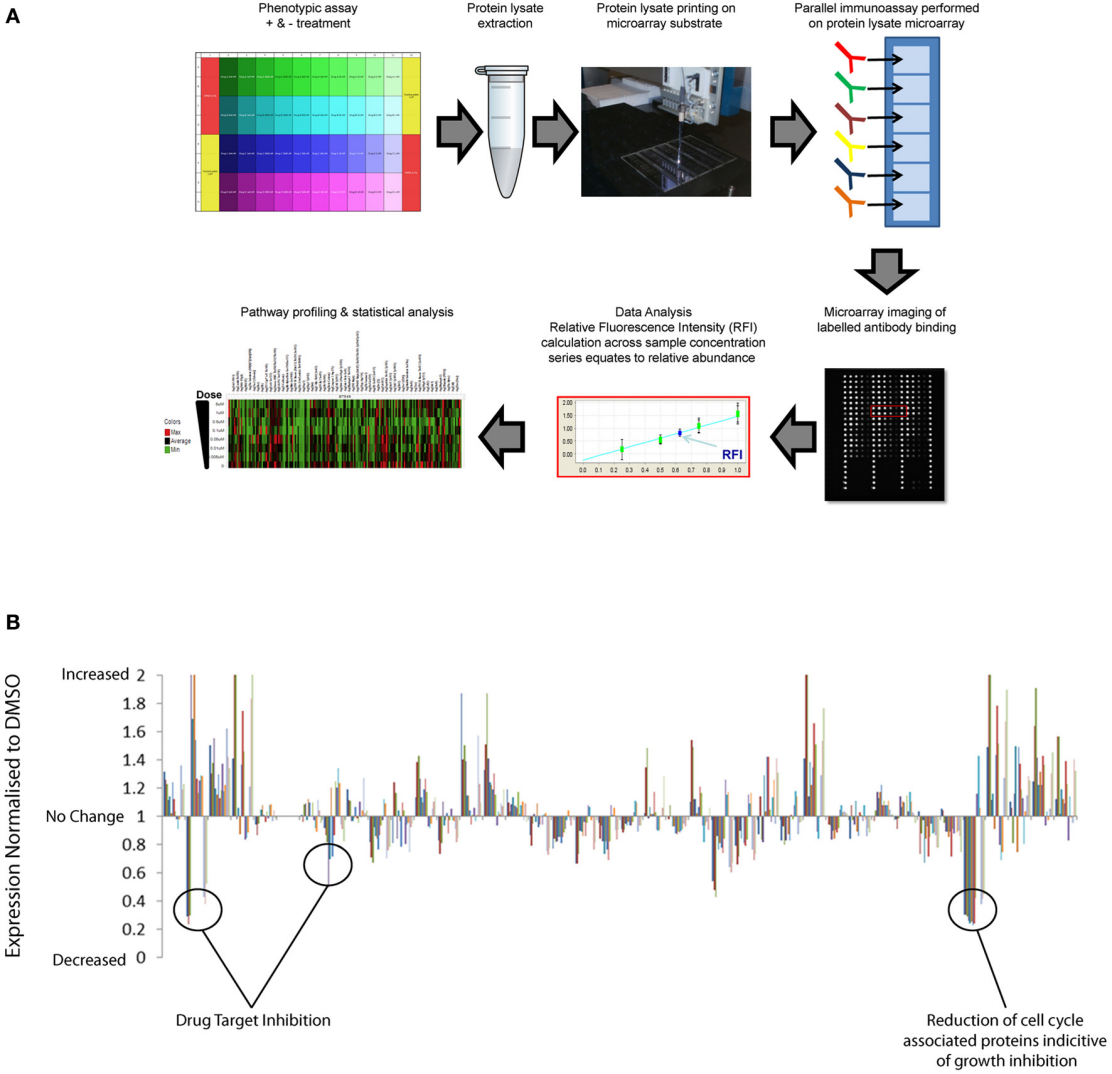
**High-throughput reverse phase protein array application to identify drug compound mechanism-of-action and compensatory response. (A)** Schematic representation of RPPA workflow: Protein lysates are extracted from multiple samples and deposited across multiple sub-arrays on a microarray by high-precision printing. Validated antibodies are individually addressed to each array, followed by incubation with labeled secondary antibody detection reagent. Antibody binding to each sample is measured by an image-based microarray scanner followed by calculation of the relative abundance of each protein and post-translational modification across the sample set. **(B)** Quantification of the relative abundance of a set of 120 total protein and post-translational pathway markers following exposure to a small set of targeted therapies across dose-response. Un-annotated pathway data normalized to respective DMSO controls demonstrate significant enhancement of multiple pathway signaling events in addition to inhibition of targeted pathways.

Traditionally, proteomics has been dependent upon quantitative mass-spectrometry techniques that remain the standard for *de novo* identification of post-translation markers. However, limitations in speed, cost, and sensitivity of mass spectrometry approaches restrict high-throughput application across multiple samples. The evolution in antibody based array methods combined with more sophisticated automation and near infrared optical detection provides new advances in sensitivity, throughput and speed of proteomics (Weissenstein et al., 2006; Voshol et al., 2009). Reverse Phase Protein Arrays (RPPAs) have previously been used to provide quantitative analysis of multiple pathway responses at the post-translational level across large numbers of biological samples simultaneously (Tibes et al., 2006; Weissenstein et al., 2006; Carey et al., 2010; Iadevaia et al., 2010). RPPA is essentially a chip and antibody-based proteomics approach to facilitate broad multiplex analysis of protein analytes, including post-translational modifications in protein extracts isolated from small samples. In Figure 1A we provide a general schematic of the RPPA procedure and refer readers to the following literature for more in-depth description of the method (Mueller et al., 2010). Pragmatic benefits of using a reverse protein array approach over alternative mass spectrometric, immunoassays, and immunohistochemical proteomic methods include:

1. Increased throughput: Sample numbers are not limited by reagent costs or instrument throughput, thereby enabling proteomic analysis of multiple compounds evaluated across dynamic dose and time—series in multiple assays and distinct model systems. Such high-throughput chemical proteomics reveal the most consistent compensatory and feedback signaling mechanisms and the genomic and physiological context in which they occur.
2. Precise and sensitive quantification of multiple pathway responses at a post-translational level, including low-abundant phophorylated-epitope signatures that can be mapped directly to drug-target hypotheses including rationale combinations. High-sensitivity enables application to small samples extracted from 96-well compound screening assays and small biopsy/Fine Needle Aspirate samples.
3. Optimal antibody multiplexing format: antibodies are physically separated on the arrays. Thus, there is no potential for antibody cross-reactivity, enabling unlimited multiplexing and optimization of concentrations and incubation buffers for every antibody.
4. Application of antibody-based detection reagents that can be readily adapted to single or small multiplex diagnostic based assays using alternative immunoassay or IHC technology.

RPPA platforms can therefore be used to simultaneously profile many pathway responses across multiple samples in high-density array formats. Pathways covered include key signaling axis, EGF receptor family, PI3K, RAS-MAPK, Src/FAK, Rb/cell-cycle, and TGF and multiple DNA repair, cell-cycle, apoptosis, and epigenetic mechanisms that have all previously been implicated in drug resistant mechanisms in cancer. Ongoing international efforts such as, NCI's Antibody Characterization Program (http://antibodies.cancer.gov); the Human Antibody Initiative (http://www.immunoportal.com/); and the Human Protein Atlas Project (www.proteinatlas.org/) to derive high quality mono-specific antibodies are poised to further advance antibody-based proteomics into broader areas of human pathway biology. Correlation of basal RPPA post-translational dataset with compound EC50 values from phenotypic assays performed across cell panels has identified protein level markers of drug sensitivity and resistance (Cardnell et al., 2013). Protein level markers of sensitivity may facilitate patient stratification to identify patient populations most likely to benefit from therapy. Protein level markers correlating with drug resistance can be mapped to drug target databases to identify rational combination strategies (Cardnell et al., 2013). In addition to monitoring the basal expression levels of total protein and post-translational markers, the high throughput nature of RPPA is ideally suited to profiling adaptive post-translational pathway network response across dose and time following compound exposure upon cells. While RPPA analysis is not compatible with live cell studies the throughput, accuracy, cost per sample, and automated nature of RPPA facilitates proteomic analysis of multiple experiments representing distinct time-points. Temporal profiling of pathway response by RPPA following drug treatment has revealed that many-targeted compounds have a dramatic effect upon reprogramming of dynamic signaling pathway networks (Lee et al., 2012). RPPA analysis of multiple pathways normalized to vehicle controls (e.g., DMSO) indicate that target inhibition can switch on many pathways as well as inhibiting others (Figure 1B). These data highlight the complexity of integrated signaling networks in living cell systems and the broad effects that targeted therapy can have upon pathway signaling. By performing RPPA studies across dose-response and time series following drug treatments, EC50 values can be calculated across multiple pathway markers over time to distinguish off-target effects from downstream signaling, feedback loop mechanisms and pathway cross talk/compensatory response. By applying RPPA to monitor the activation state of pathways, compensatory signaling response can be directly mapped to drug-target databases to build rational drug combination strategies that can be validated across phenotypic assays or preclinical models.

### Concluding remarks

High-content imaging is leveraging functional phenotypic endpoints from more complex assay formats such as multicellular co-culture and 3D models, which are ideally suited for screening multi-targeted agents and drug combinations. An attraction of phenotypic screening across panels of human cells derived from patient's disease or human induced pluripotent stem cell (IPSC) models is correlation of phenotypic response with genomic biomarkers. Such pharmacogenomic studies support biomarker discovery and patient stratification hypothesis. However, as described in this review article, adaptive resistance mechanisms that guide combination response are often operating only at the post-translational level. Thus, RPPA analysis applied to related monotherapy and drug combination arms can be correlated with additive, synergistic, or antagonist phenotypic response across a dose matrix study to identify pharmacodynamic biomarkers that confirm the mechanism-of-action of the drug combination effect and provide biomarkers to guide future clinical development.

In 2010, the US FDA issued an updated draft guidance to support the further development of novel drug-combination therapies. Previous guidance recommended demonstrative evidence of a positive efficacy and safety response across all monotherapy and combination arms. The updated guidance now supports the proposal of clinical trial designs where single or multiple monotherapy arms can be left out on the grounds that no efficacy benefit would be expected. This new update now provides an opportunity to exploit synthetic lethality and multi-drug cocktails. Phenotypic screening campaigns are particularly suited to discovery of synthetic lethality by performing screens on matched pairs of cell models, representing suspected natural or engineered genetic vulnerabilities or distinct sensitivities to known agents. The latest advances in phenotypic screening technologies combined with high-throughput pathway profiling are now well placed to provide high quality preclinical data that provide for a more robust, transparent and objective prioritization of drug combinations.

## Author contributions

Both authors contributed to the conception of this review article, drafted the paper, and approved the version for submission.

### Conflict of interest statement

The authors declare that the research was conducted in the absence of any commercial or financial relationships that could be construed as a potential conflict of interest.

## References

[B1] Al-Lazikani B. Banerji U. Workman P. (2012). Combinatorial drug therapy for cancer in the post-genomic era. Nat. Biotechnol. 30, 679–692 10.1038/nbt.2284 22781697

[B2] Alcock P. Bath C. Blackett C. Simpson P. B. (2010). High content cell based primary screening for oncology targets – a perspective. Eur. Pharm. Rev. 3

[B3] Axelrod M. Gordon V. L. Conaway M. Tarcsafalvi A. Neitzke D. J. Gioeli D. (2013). Combinatorial drug screening identifies compensatory pathway interactions and adaptive resistance mechanisms. Oncotarget 4, 622–635 23599172 10.18632/oncotarget.938 PMC3720609

[B4] Azmi A. S. Wang Z. Philip P. A. Mohammad R. M. Sarkar F. H. (2010). Proof of concept: network and systems biology approaches aid in the discovery of potent anticancer drug combinations. Mol. Cancer Ther. 9, 3137–3144 10.1158/1535-7163.MCT-10-0642 21041384 PMC3058926

[B5] Barretina J. Caponigro G. Stransky N. Venkatesan K. Margolin A. A. Kim S. (2012). The Cancer cell line encyclopedia enables predictive modelling of anticancer drug sensitivity. Nature 483, 603–607 10.1038/nature11003 22460905 PMC3320027

[B6] Bickle M. (2008). High-content screening: a new primary screening tool? IDrugs 11, 822–826 18988127

[B7] Bickle M. (2010). The beautiful cell: high-content screening in drug discovery. Anal. Bioanal. Chem. 398, 219–226 10.1007/s00216-010-3788-3 20577725

[B8] Caie P. D. Walls R. E. Ingleston-Orme A. Daya S. Houslay T. Eagle R. (2010). High-content phenotypic profiling of drug response signatures across distinct cancer cells. Mol. Cancer Ther. 9, 1913–1926 10.1158/1535-7163.MCT-09-1148 20530715

[B9] Cardnell R. J. Feng Y. Diao L. Fan Y. H. Masrorpour F. Wang J. (2013). Proteomic markers of DNA repair and PI3K pathway activation predict response to the PARP inhibitor BMN 673 in small cell lung cancer. Clin. Cancer Res. 19, 6322–6328 10.1158/1078-0432.CCR-13-1975 24077350 PMC3882158

[B10] Carey M. S. Agarwal R. Gilks B. Swenerton K. Kalloger S. Santos J. (2010). Functional proteomic analysis of advanced serous ovarian cancer using reverse phase protein array: TGF-beta pathway signaling indicates response to primary chemotherapy. Clin. Cancer Res. 16, 2852–2860 10.1158/1078-0432.CCR-09-2502 20460476 PMC2877659

[B11] Chou T. C. Talalay P. (1981). Generalized equations for the analysis of inhibitions of Michaelis-Menten and higher-order kinetic systems with two or more mutually exclusive and nonexclusive inhibitors. Eur. J. Biochem. 115, 207–216 10.1111/j.1432-1033.1981.tb06218.x 7227366

[B12] Chou T. C. Talalay P. (1984). Quantitative analysis of dose-effect relationships: the combined effects of multiple drugs or enzyme inhibitors. Adv. Enzyme Regul. 22, 27–55 10.1016/0065-2571(84)90007-4 6382953

[B13] Cubitt C. L. Menth J. Dawson J. Martinez G. V. Foroutan P. Morse D. L. (2013). Rapid screening of novel agents for combination therapy in sarcomas. Sarcoma 2013:365723 10.1155/2013/365723 24282374 PMC3824404

[B14] Dabhadkar K. C. Bellam N. (2013). Polypill strategy for primary prevention of cardiovascular disorders. Drugs Today 49, 317–324 10.1358/dot.2013.49.5.1950148 23724411

[B15] Du G. S. Pan J. Z. Zhao S. P. Zhu Y. Den Toonder J. M. Fang Q. (2013). Cell-based drug combination screening with a microfluidic droplet array system. Anal. Chem. 85, 6740–6747 10.1021/ac400688f 23786644

[B16] Duncan J. S. Whittle M. C. Nakamura K. Abell A. N. Midland A. A. Zawistowski J. S. (2012). Dynamic reprogramming of the kinome in response to targeted MEK inhibition in triple-negative breast cancer. Cell 149, 307–321 10.1016/j.cell.2012.02.053 22500798 PMC3328787

[B17] Flaherty K. T. Infante J. R. Daud A. Gonzalez R. Kefford R. F. Sosman J. (2012). Combined BRAF and MEK inhibition in melanoma with BRAF V600 mutations. N. Engl. J. Med. 367, 1694–1703 10.1056/NEJMoa1210093 23020132 PMC3549295

[B18] Harma V. Virtanen J. Makela R. Happonen A. Mpindi J. P. Knuuttila M. (2010). A comprehensive panel of three-dimensional models for studies of prostate cancer growth, invasion and drug responses. PLoS ONE 5:e10431 10.1371/journal.pone.0010431 20454659 PMC2862707

[B19] Held M. A. Langdon C. G. Platt J. T. Graham-Steed T. Liu Z. Chakraborty A. (2013). Genotype-selective combination therapies for melanoma identified by high-throughput drug screening. Cancer Discov. 3, 52–67 10.1158/2159-8290.CD-12-0408 23239741 PMC3546137

[B20] Iadevaia S. Lu Y. Morales F. C. Mills G. B. Ram P. T. (2010). Identification of optimal drug combinations targeting cellular networks: integrating phospho-proteomics and computational network analysis. Cancer Res. 70, 6704–6714 10.1158/0008-5472.CAN-10-0460 20643779 PMC2932856

[B21] Jabbour S. A. Hardy E. Sugg J. Parikh S. (2014). Dapagliflozin is effective as add-on therapy to sitagliptin with or without metformin: A 24-week, multicenter, randomized, double-blind, placebo-controlled study. Diabetes Care 37, 740–750 10.2337/dc13-0467 24144654

[B22] Kolch W. Pitt A. (2010). Functional proteomics to dissect tyrosine kinase signalling pathways in cancer. Nat. Rev. Cancer 10, 618–629 10.1038/nrc2900 20720570

[B23] Lee J. A. Berg E. L. (2013). Neoclassic drug discovery: the case for lead generation using phenotypic and functional approaches. J. Biomol. Screen. 18, 1143–1155 10.1177/1087057113506118 24080259

[B24] Lee M. J. Ye A. S. Gardino A. K. Heijink A. M. Sorger P. K. Macbeath G. (2012). Sequential application of anticancer drugs enhances cell death by rewiring apoptotic signaling networks. Cell 149, 780–794 10.1016/j.cell.2012.03.031 22579283 PMC3501264

[B25] Lehar J. Krueger A. S. Avery W. Heilbut A. M. Johansen L. M. Price E. R. (2009). Synergistic drug combinations tend to improve therapeutically relevant selectivity. Nat. Biotechnol. 27, 659–666 10.1038/nbt.1549 19581876 PMC2708317

[B26] Li X. Tong L. J. Ding J. Meng L. H. (2014). Systematic combination screening reveals synergism between rapamycin and sunitinib against human lung cancer. Cancer Lett. 342, 159–166 10.1016/j.canlet.2013.08.046 24018642

[B27] Mueller C. Liotta L. A. Espina V. (2010). Reverse phase protein microarrays advance to use in clinical trials. Mol. Oncol. 4, 461–481 10.1016/j.molonc.2010.09.003 20974554 PMC2981612

[B28] Oikonomopoulos G. M. Syrigos K. N. Skoura E. Saif M. W. (2014). FOLFIRINOX: from the ACCORD study to 2014. JOP 15, 103–105 10.6092/1590-8577/2278 24618428

[B29] Perlman Z. E. Slack M. D. Feng Y. Mitchison T. J. Wu L. F. Altschuler S. J. (2004). Multidimensional drug profiling by automated microscopy. Science 306, 1194–1198 10.1126/science.1100709 15539606

[B30] Sacks L. V. Behrman R. E. (2009). Challenges, successes and hopes in the development of novel TB therapeutics. Future Med. Chem. 1, 749–756 10.4155/fmc.09.53 21426037

[B31] Sahoo R. K. Kumar L. (2014). Albumin-bound paclitaxel plus gemcitabine in pancreatic cancer. N. Engl. J. Med. 370, 478–479 10.1056/NEJMc1314761 24476440

[B32] Saltz L. B. Bach P. B. (2014). Albumin-bound paclitaxel plus gemcitabine in pancreatic cancer. N. Engl. J. Med. 370:478 10.1056/NEJMc1314761 24476439

[B33] Schmidt L. Kling T. Monsefi N. Olsson M. Hansson C. Baskaran S. (2013). Comparative drug pair screening across multiple glioblastoma cell lines reveals novel drug-drug interactions. Neuro Oncol. 15, 1469–1478 10.1093/neuonc/not111 24101737 PMC3813417

[B34] Smith G. S. Voyer-Grant J. A. Harauz G. (2012). Monitoring cleaved caspase-3 activity and apoptosis of immortalized oligodendroglial cells using live-cell imaging and cleaveable fluorogenic-dye substrates following potassium-induced membrane depolarization. J. Vis. Exp. e3422 10.3791/3422 22294086 PMC3462581

[B35] Stommel J. M. Kimmelman A. C. Ying H. Nabioullin R. Ponugoti A. H. Wiedemeyer R. (2007). Coactivation of receptor tyrosine kinases affects the response of tumor cells to targeted therapies. Science 318, 287–290 10.1126/science.1142946 17872411

[B36] Tanaka M. Bateman R. Rauh D. Vaisberg E. Ramachandani S. Zhang C. (2005). An unbiased cell morphology-based screen for new, biologically active small molecules. PLoS Biol. 3:e128 10.1371/journal.pbio.0030128 15799708 PMC1073692

[B37] Tibes R. Qiu Y. Lu Y. Hennessy B. Andreeff M. Mills G. B. (2006). Reverse phase protein array: validation of a novel proteomic technology and utility for analysis of primary leukemia specimens and hematopoietic stem cells. Mol. Cancer Ther. 5, 2512–2521 10.1158/1535-7163.MCT-06-0334 17041095

[B38] Von Hoff D. D. Goldstein D. Renschler M. F. (2014). Albumin-bound paclitaxel plus gemcitabine in pancreatic cancer. N. Engl. J. Med. 370, 479–480 10.1056/NEJMc1314761 24476438

[B39] Voshol H. Ehrat M. Traenkle J. Bertrand E. Van Oostrum J. (2009). Antibody-based proteomics: analysis of signaling networks using reverse protein arrays. FEBS J. 276, 6871–6879 10.1111/j.1742-4658.2009.07395.x 19860827

[B40] Weissenstein U. Schneider M. J. Pawlak M. Cicenas J. Eppenberger-Castori S. Oroszlan P. (2006). Protein chip based miniaturized assay for the simultaneous quantitative monitoring of cancer biomarkers in tissue extracts. Proteomics 6, 1427–1436 10.1002/pmic.200500078 16440370

[B41] Yap T. A. Omlin A. De Bono J. S. (2013). Development of therapeutic combinations targeting major cancer signaling pathways. J. Clin. Oncol. 31, 1592–1605 10.1200/JCO.2011.37.6418 23509311

[B42] Yarrow J. C. Perlman Z. E. Westwood N. J. Mitchison T. J. (2004). A high-throughput cell migration assay using scratch wound healing, a comparison of image-based readout methods. BMC Biotechnol. 4:21 10.1186/1472-6750-4-21 15357872 PMC521074

[B43] Zimmermann G. R. Lehar J. Keith C. T. (2007). Multi-target therapeutics: when the whole is greater than the sum of the parts. Drug Discov. Today 12, 34–42 10.1016/j.drudis.2006.11.008 17198971

[B44] Zinner R. G. Barrett B. L. Popova E. Damien P. Volgin A. Y. Gelovani J. G. (2009). Algorithmic guided screening of drug combinations of arbitrary size for activity against cancer cells. Mol. Cancer Ther. 8, 521–532 10.1158/1535-7163.MCT-08-0937 19276160

